# Staple Containing Ureteral Stone Formation After Robot-Assisted Radical Cystectomy With Intracorporeal Neobladder Construction in a Female Patient: A Case Report of a Rare Complication

**DOI:** 10.7759/cureus.27712

**Published:** 2022-08-05

**Authors:** Nikolaos Liakos, Mikolaj A Mendrek, Theodoros Karagiotis, Sami-Ramzi Leyh-Bannurah, Joern Witt

**Affiliations:** 1 Department of Urology, Pediatric Urology and Urological Oncology, St. Antonius Hospital Gronau, Gronau, DEU

**Keywords:** surgical staples, robot-assisted cystectomy, complication, neobladder, ureteral stone

## Abstract

Radical cystectomy with intracorporeal neobladder formation is a well-established treatment for patients with muscle-invasive urothelial cancer of the bladder. After the wide implementation of robotic systems, numerous centers increasingly offer intracorporeal neobladder construction using robotic staple devices. Stone formation at the area of staple material migration is a long-term complication, as staple material may migrate through the neobladder wall and act as a nidus for urine crystal aggregation. Stone formation in the upper urinary tract and the diversion segment is highly variable and corresponding management protocols are extensively reported in the previous series. However, the presence of staple material within a renal or ureteral stone has been rarely reported before. We present a case of a female patient with a staple-containing ureteral stone four years after radical cystectomy and neobladder formation and the consequent antegrade ureterorenoscopic retrieval. This treatment option is feasible, safe and easily implemented by experienced endourologists.

## Introduction

Radical cystectomy with neobladder reconstruction is a well-established treatment for patients with localized urothelial bladder cancer, given a life expectancy of at least 10 years and a sufficient performance status [1]. Among major urological surgeries, radical cystectomy represents the procedure associated with the highest morbidity and mortality rates [2]. Expectedly, complication rates in high-volume tertiary centers are lower than in low-volume centers. Thus, referring these patients to experienced centers is an effective measure of reducing peri- and postoperative complications.

Complications may be categorized according to the chronology, i.e. onset after surgery (short-, intermediate- and long-term) or classified as, e.g. genitourinary, gastrointestinal, metabolic, infectious, pulmonary or lymphatic complications [3]. Here, genitourinary complications include the formation of stones in the lower urinary system. Such stone formation predominantly occurs in patients with a neobladder diversion, but cases have been also reported in those with non-orthotopic urinary diversion. Specifically, the occurrence of ureteral stones is rather infrequent, with a rate up to 3.5% [3]. Their treatment may be conservative (with spontaneous passage) or minimal-invasive, such as applying endourological stone extraction. As there are numerous variants of ureterointestinal anastomosis [4] and neobladder formation, the ureterorenoscopic procedures may vary from easy to highly challenging. Here, we present a rare case of a staple-containing ureteral stone after robot-assisted radical cystectomy with intracorporeal neobladder construction using a robotic stapling device, with a challenging stone extraction process.

## Case presentation

A 52-year-old female patient was diagnosed with muscle-invasive urothelial bladder cancer (final pathology: pT2 pN0 cM0 high grade, negative surgical margins) in 2016. Further relevant medical history included chronic hepatitis and non-Hodgkin lymphoma, which was classified as a complete response after completing chemotherapy.

Seven weeks after diagnosis, she underwent a robot-assisted vagina-sparing radical cystectomy with intracorporeal orthotopic urinary diversion, receiving a modified U-shaped ileal neobladder with right-sided chimney construction and right lateral refluxing ureteroileal anastomosis with side-to-side ureteroureteral anastomosis (Wallace technique). This technique was first described by the Department of Urology at Karolinska University [5]. Due to postoperative neobladder emptying disturbances with high post voiding residual urine volume (greater than 500ml) after the first postoperative year, a suprapubic neobladder catheter was inserted for continuous urine drainage and changed in intervals of four to six weeks. This was well tolerated by the patient.

**Figure 1:**
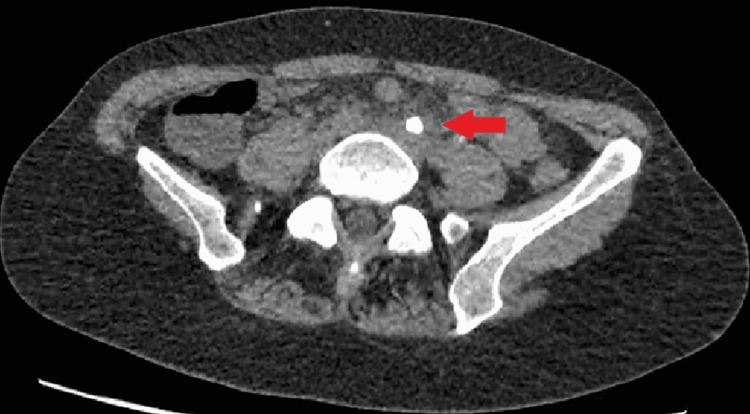
CT-abdomen, native sequence, ureteral stone in the medial portion of the left ureter (6-mm diameter, 1100 Hounsfield units), axial view.

**Figure 2:**
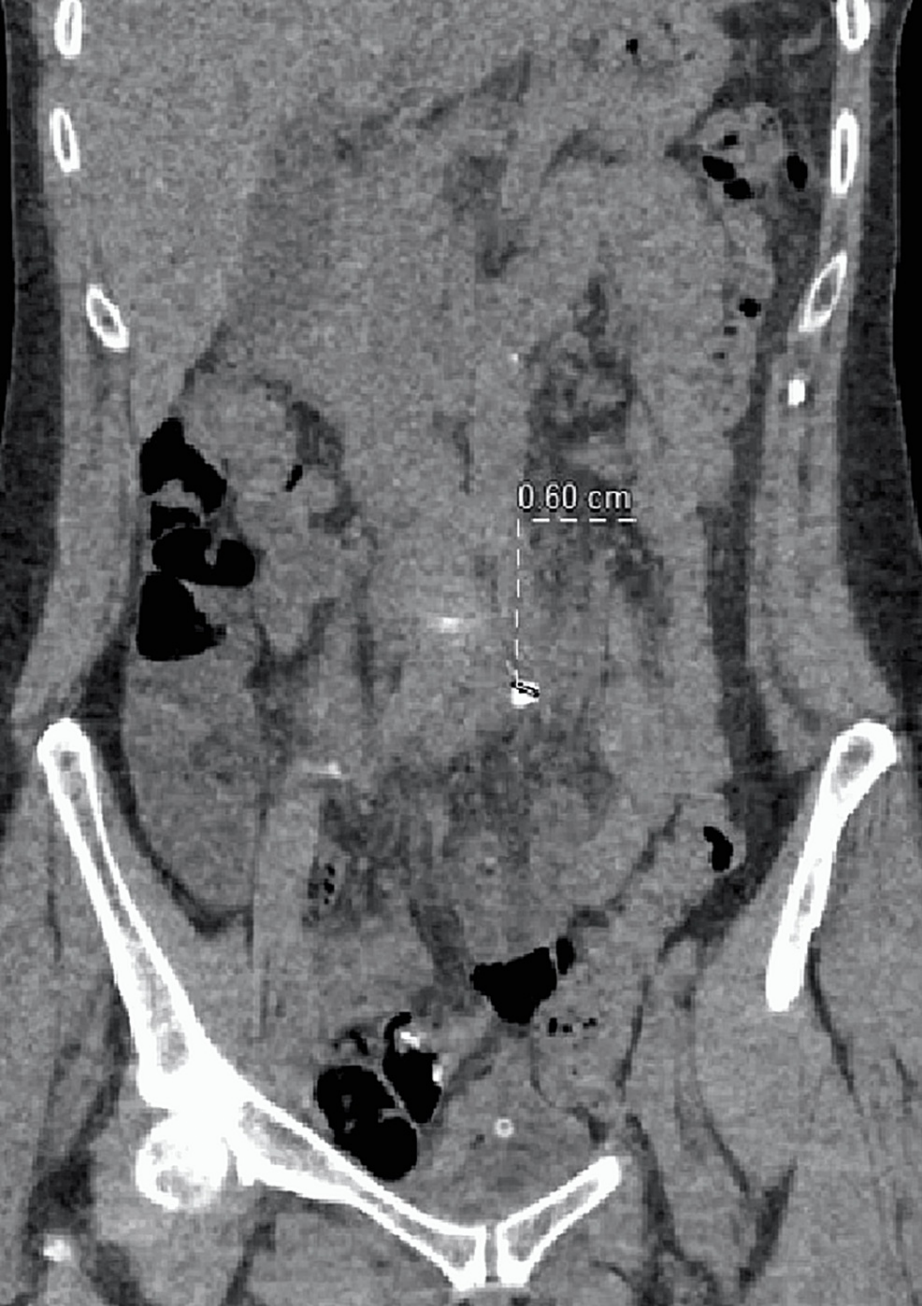
CT-abdomen, native sequence, ureteral stone in the medial portion of the left ureter (6-mm, 1100 Hounsfield units), coronal view.

The patient presented in our department 49 months after our surgery with acute flank pain on the left side. Ultrasonographically, a grade two dilatation of the left pelvic system was confirmed. After ensuring adequate kidney function (creatinine 1.18 mg/dL, eGFR 48.06 mL/min/1.73m²) and due to the initial presumption, ureteroileal stenosis might be the reason for the hydronephrosis, a CT urography was conducted (Figures 1, 2). A 6-mm left ureteral stone (1100 Hounsfield units) on the middle part of the ureter could be identified. As the first measure, an ultrasound-guided percutaneous nephrostomy was inserted on the left side to relieve patient symptoms and secure urine diversion. The stone extraction was planned for three weeks thereafter.

**Figure 3:**
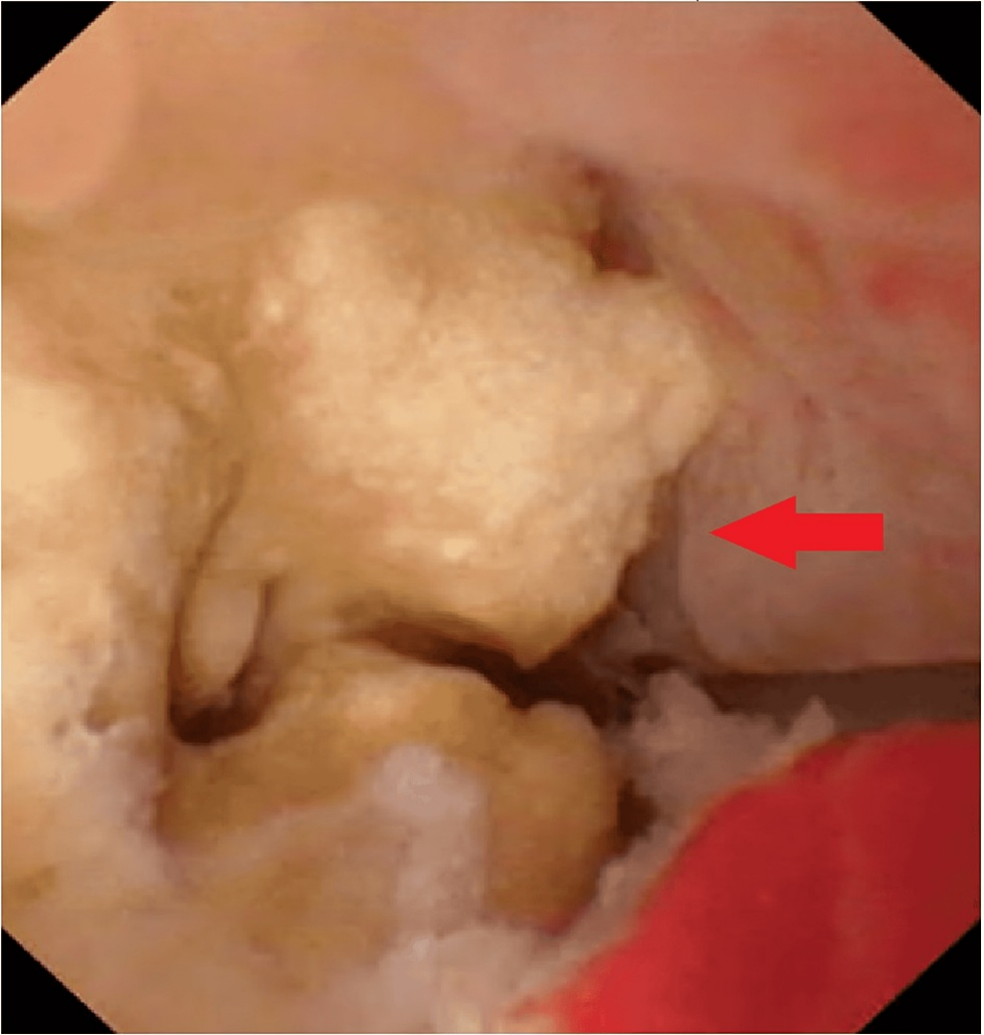
Endoscopic view of the stone during left side antegrade flexible ureterorenoscopy.

Placing the patient in the prone position and under general anesthesia, the initial puncture canal was dilated and a ureteral sheath (10 french) was placed in an antegrade manner. Using a flexible ureterorenoscope, we proceeded to the antegrade endoscopic evaluation of the left upper urinary tract. The ureteral stone was found in the middle third of the left ureter (Figure 3). Due to the stone dimension, we proceeded to the lithotripsy using a Holmium laser fiber (settings unfortunately not documented). A portion of the stone was extracted for further chemical analysis and the rest was “dusted.” During the dusting procedure, we identified a metallic object within the stone. After its extraction, we discovered that this metallic object was a non-absorbable staple from the robotic stapler (titan non-absorbable staples, Endowrist Stapler for the Da Vinci XI™ robotic system, Sunnyvale, CA, USA), used during the ileal isolation and consequent formation of the neobladder. The subsequent endoscopic evaluation of the left upper tract showed a stone-free situation and further endoscopic treatment of the patient was uneventful. After the extraction of the ureteral sheath, a pigtail catheter was placed in the ureter and the catheter extraction followed two weeks after urolithiasis treatment.

As the intraurethral presence of the staple could not be justified and a staple migration could not be explained, we proceeded to the comprehensive video analysis of the neobladder reconstruction during the robot-assisted radical cystectomy in our institutional morbidity and mortality conference, as the sequence was saved in our video databank. All extraneovesical staple material, apart from these ensuring a sufficient closure of the vessels of the mesentery, was meticulously removed during the surgery. The location of the ureteral stone was above the height of the mesocolic incision for the translocation of the contralateral ureter, almost excluding the possibility of a staple migration in the retroperitoneum. 

The only possible way to explain the migration of the staple into the upper urinary tract and allowing a stone formation is an unidentified passage of a remaining staple within the upper urinary tract during the retrograde splint placement into the ureters and the kidney pelvis during the reconstructive phase of the surgery, a surgical step before the formation of the ureteroileal anastomosis. We could not identify any staple misplacement into the left ureter from the anterior side during the video analysis. Thus, we can only presume that we accidentally moved this singular, dorsal-lying staple upward the urinary tract and misplaced it into the kidney pelvis. Moreover, the formation of a 6-mm renal stone around the staple is conceivable within the time period of 49 months. As the staple-containing stone passed through the ureteropelvic junction and after occluding the medial portion of the left ureter due to its dimensions, symptomatic hydronephrosis was the result.

## Discussion

The formation of urinary stones is one of the various postoperative long-term complications after radical cystectomy and urinary diversion. Due to diversion-associated changes in urinary metabolic effects, as well as complex changes in urinary stasis and reflux, a neobladder diversion may be associated with stone formation in about 5% of the patients [3]. The preferred treatment in a case with neobladder stone formation is a transurethral or percutaneous stone extraction [6-9], whereas the cases presenting stones in the upper urinary tract may be quite challenging for the attending urologists. There are many access strategies and approaches reported in the literature. The most favorable outcomes are the antegrade ureterorenoscopic approach for ureteral stones and percutaneous nephrolitholapaxy (PCNL) for kidney stones in need of treatment [10-13]. El-Assmy et al. described treating kidney stones after neobladder construction by extracorporal shock wave lithotripsy (ESWL) [14], but this study had a limited sample size and there have been no further studies evaluating this approach.

The retrograde ureterorenoscopic approach is the treatment of choice by patients with an ileal conduit due to the brief distance to the stoma orifice, though the management of ureteral stones by patients with neobladder can be a challenge for the attending urologist due to the complex anatomy after surgery. The study of Zhong et al. [11] concludes that antegrade access is superior to retrograde ureterorenoscopy due to unsuccessful and difficult efforts to identify the neovesicoureteral orifice. Moreover, potential relative stenosis of the ureteroneovesical junction must be considered. Thus, the choice of an antegrade approach is favored by many urologists. 

To the best of our knowledge, there is only one case report of clip migration after radical cystectomy into the upper urinary tract by Albadawi et al. [15], but there is no mention in the international literature about staple migration with the metachronous stone formation in the upper urinary tract.

By presenting this case, we present a treatment approach to this challenging scenario by implementing an antegrade access path to the middle portion of the ureter. We showed that the antegrade flexible ureterorenoscopy with subsequent laser lithotripsy and staple material harvesting is a feasible option. Through this specific approach, there is a sparing of any possible endoscopic manipulations in the ureteroneovesical anastomosis, which may cause a stricture of the anastomosis and could have a detrimental effect on the future urinary drainage to the neobladder. Our proposed surgical option is safe, time- and tissue-sparing and can be easily implemented by experienced endourologists. As a take home message, we would like to suggest that patients presenting with symptoms suggestive of acute renal colic and previously undergoing major urologic surgery should be thoroughly examined for staple- or clip-related complications, as seen in previously published studies [16]. In addition, urologists need to remember that an expedited intervention may be required.

## Conclusions

Although stone formation is mainly identified in the neobladder after radical cystectomy with neobladder formation, stone formation in the upper urinary tract cannot be excluded. Moreover, material migration after robot-assisted cystectomy and stone formation mostly affects the neobladder wall and the intraneovesical space. The possibility of intraluminal migration of staple material up to the kidney pelvis, as in our case report, is extremely rare. The antegrade ureterorenoscopic approach is a safe extraction method of the migrated material, preserving the integrity of the ureteroneovesical anastomosis and offering a treatment option without increasing the possibility of further complications during and after endoscopic surgery. It is of utmost importance to prevent any accidental misplacement of free staple material during the various surgical steps during the reconstructive phase. Our surgical approach is highly consistent with other, similar scenarios presented in the international literature.
